# Genome Sequences of Bacteria Associated with the Diatom *Cyclotella cryptica* Strain CCMP332

**DOI:** 10.1128/MRA.01030-20

**Published:** 2020-11-12

**Authors:** Cory B. Gargas, Wade R. Roberts, Andrew J. Alverson

**Affiliations:** a Department of Biological Sciences, University of Arkansas, Fayetteville, Arkansas, USA; University of Delaware

## Abstract

We report draft genomes of two bacterial strains in the genera *Hyphobacterium* and *Reichenbachiella*, which are associated with the diatom *Cyclotella cryptica* strain CCMP332. Genomes from these strains were 2,691,501 bp and 3,325,829 bp, respectively, and will be useful for understanding interactions between diatoms and bacteria.

## Abstract (precis)

We report draft genomes of two bacterial strains in the genera *Hyphobacterium* and *Reichenbachiella*, which are associated with the diatom *Cyclotella cryptica* strain CCMP332. Genomes from these strains were 2,691,501 bp and 3,325,829 bp in size, respectively, and will be useful for understanding interactions between diatoms and bacteria.

## ANNOUNCEMENT

The phycosphere (1) describes the consortium of bacteria associated with diatoms and other algae. We assembled and annotated draft genomes from two bacteria associated with *Cyclotella cryptica* strain CCMP332, a diatom of longstanding interest for its biology and promising biotechnological applications (2, 3).

We acquired strain CCMP332 from the National Center for Marine Algae and Microbiota. CCMP332 was originally isolated in 1956 in Martha's Vineyard, Massachusetts, by R. Guillard. We grew CCMP332 in L1 medium (4) at 22°C on a 12-h/12-h light/dark cycle. We harvested cells by centrifugation and extracted DNA using the cetyltrimethylammonium bromide (CTAB) protocol (5). We prepared one sequencing library using the Oxford Nanopore Technologies (ONT) ligation sequencing kit (SQK-LSK108) for sequencing on the ONT MinION platform. We prepared short-read Illumina libraries using the Kapa HyperPlus kit (Roche) with 300- to 400-bp insert sizes for sequencing on the Illumina HiSeq 4000 platform.

**FIG 1:**
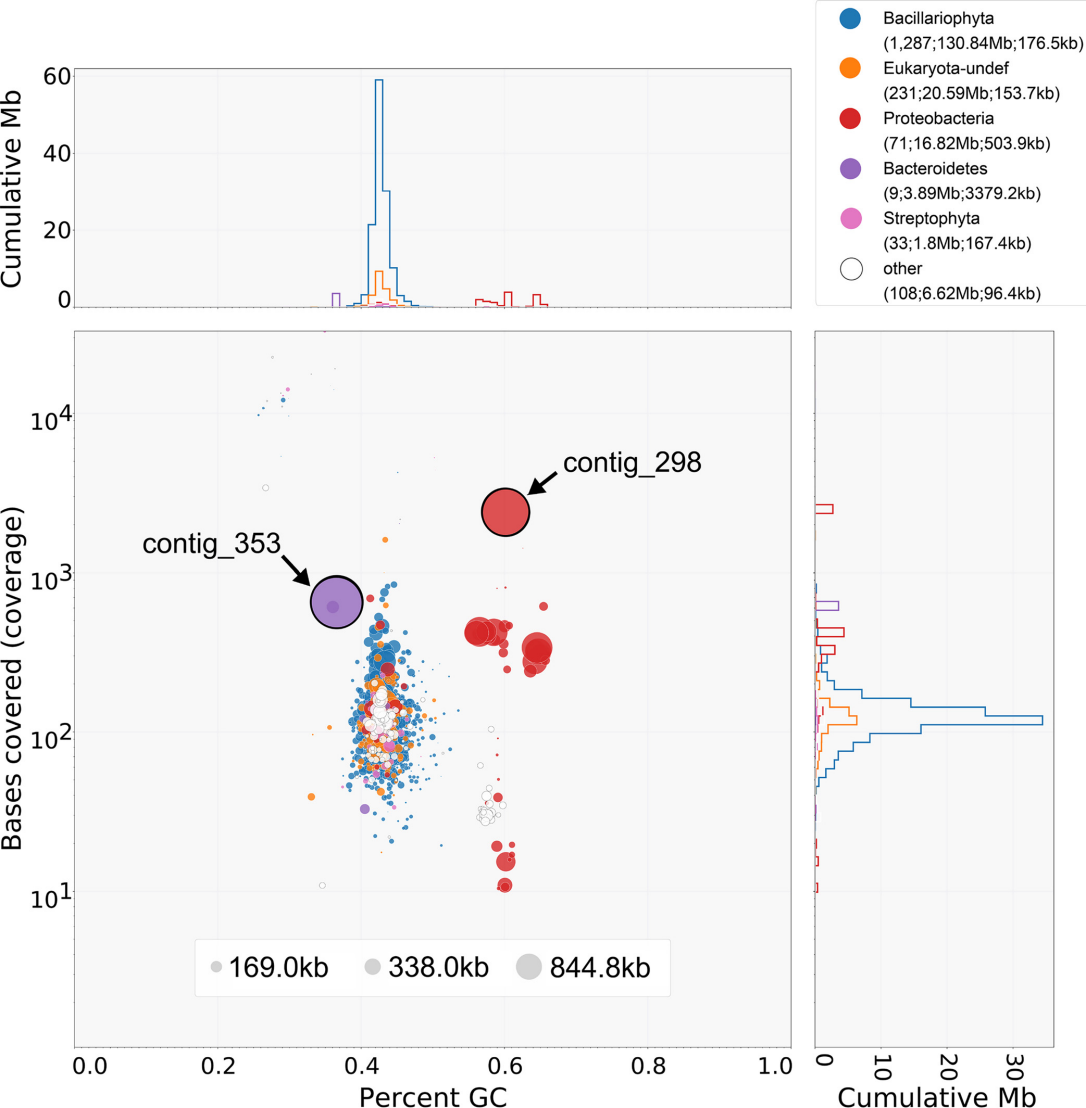
Blob plot showing the taxon-annotated GC content and read coverage of the assembly contigs. Each circle represents one contig, and the circle size corresponds to the contig length (in base pairs). Colors correspond to the taxonomic assignment of each contig via BLASTX searches against the UniProt Reference Proteomes database. Histograms along each axis show the cumulative megabases for each phylum. Read coverage was calculated from the alignment of both MinION and Illumina reads. The circles corresponding to contigs 298 and 353 are labeled and outlined. The data shown in the key have the following format: phylum (number of contigs; length of contigs; contig *N*_50_ value).

For all analyses, default software parameters were used unless noted otherwise. We used Guppy (v.2.3.5) for base calling of the MinION reads and Canu (v.1.7) (6) for error correction of the raw MinION reads. We assembled the raw MinION reads with Flye (v.2.4.2, using the parameter --genome-size 165m) (7) and visualized the assembly with BlobTools (v.1.1.1) (8) to identify the bacterial genomes (Fig. 1). We mapped the corrected MinION reads to draft contigs with minimap2 (v.2.10-r761) (9) for contig correction with Racon (v.1.3.3) (10). We then mapped the Illumina reads to the contigs with BWA-MEM (v.0.7.17-r1188) (11) and performed three rounds of contig polishing with Pilon (v.1.2.2, using the parameter “fix bases”) (12). We confirmed the circularity of contig 298 with Circlator (v.1.5.5) (13) but could not circularize contig 353. We used Prokka (v.1.14.6) (14) for genome annotation and EPA-NG (v.0.3.5) (15) and Gappa (v.0.5.0) (16) for phylotaxonomic placement of each genome. Genome completeness was assessed at each assembly stage with benchmarking universal single-copy orthologs (BUSCO) (v.4.0.6, using the alphaproteobacteria_odb10 and bacteroidetes_odb10 data sets) (17).

Draft contig correction and polishing increased BUSCO completeness to 94.6% and 98.0% for contigs 298 and 353, respectively. The characteristics and annotation summaries for each genome are listed in Table 1. Contig 298 is closely related to an undescribed species of *Hyphobacterium* (*Alphaproteobacteria*). This assignment is plausible based on known environmental preferences of other *Hyphobacterium* species (18, 19). Two 16S rRNA genes in contig 353 were 98.1% identical, and both were placed in the family *Cyclobacteriaceae*, with one assigned only at the family level and the other assigned to the genus *Reichenbachiella. Reichenbachiella* species live in marine habitats (20–23), and one species has been associated with the red alga *Gracilaria blodgettii* (23). Low support for the placement of contig 353 in *Reichenbachiella* might indicate that the genome belongs to an undescribed genus that is closely related to *Reichenbachiella*.

**TABLE 1:** Characteristics, annotations, and taxonomy of bacterial genomes associated with *C. cryptica* CCMP332

Characteristic	Genome data for:	
	Contig 298	Contig 353
GenBank accession no.	CP058669	CP058647
GC content (%)	60.15	37.09
Avg read coverage (×)	1,225	464
Circularity	Confirmed^*a*^	Linear
Genome size (bp)	2,690,927	3,325,829
No. of long reads^*b*^	11,448	25,404
No. of short reads (single/paired)^*c*^	52,515,998/22,560,574	6,305,304/18,014,964
Bases confirmed by Illumina (%)	99.63	99.94
Complete BUSCO count (%)	397 (94.6)^*d*^	394 (98.0)^*e*^
No. of CDS^*f*^ (functional/hypothetical)	1,394/1,207	1,310/1,629
No. of pseudogenes	56	12
No. of tRNAs	103	37
No. of tmRNAs	1	1
EPA-NG taxonomy^*g*^	*Hyphobacterium* sp. 002783385	*Cyclobacteriaceae*/*Reichenbachiella* sp.
EPA-NG LWR^*g*^^,^^*h*^	86.84	65.57/49.99

a “Confirmed” indicates that both Flye and Circlator confirmed circularity of a genome.

b Out of 5,951,322 total MinION reads. MinION *N*_50_, 4,395 bp.

c Out of 449,895,635 total Illumina reads.

d Genome mode against the alphaproteobacteria_odb10 data set.

e Genome mode against the bacteroidetes_odb10 data set.

f CDS, coding sequences.

g Multiple values or taxonomic names indicate differences in taxonomic assignment or LWR values between 16S copies.

h Length weight ratio (LWR) is a support value for EPA-NG phylogenetic assignments; higher values indicate higher support for that assignment.

### Data availability.

Genome sequences have been deposited in GenBank under accession numbers CP058669 and CP058647, as part of BioProject PRJNA642781. Sequencing reads are available under BioProject PRJNA628076. Please note that the *Cyclobacteriaceae* sp. is listed on NCBI as a *Hyphobacterium* sp.

## References

[1] Seymour JR, Amin SA, Raina J-B, Stocker R 2017 Zooming in on the phycosphere: the ecological interface for phytoplankton-bacteria relationships. Nat Microbiol 2:17065. doi:10.1038/nmicrobiol.2017.65. 28555622

[2] Traller JC, Cokus SJ, Lopez DA, Gaidarenko O, Smith SR, McCrow JP, Gallaher SD, Podell S, Thompson M, Cook O, Morselli M, Jaroszewicz A, Allen EE, Allen AE, Merchant SS, Pellegrini M, Hildebrand M 2016 Genome and methylome of the oleaginous diatom *Cyclotella cryptica* reveal genetic flexibility toward a high lipid phenotype. Biotechnol Biofuels 9:258. doi:10.1186/s13068-016-0670-3. 27933100 PMC5124317

[3] Roberts WR, Downey KM, Ruck EC, Traller JC, Alverson AJ 2020 Improved reference genome for *Cyclotella cryptica* CCMP332, a model for cell wall morphogenesis, salinity adaptation, and lipid production in diatoms (Bacillariophyta). G3 (Bethesda) 10:2965–2974. doi:10.1534/g3.120.401408. 32709619 PMC7466962

[4] Guillard RRL 1975 Culture of phytoplankton for feeding marine invertebrates, p 29–60. *In* Smith WL, Chanley MH (ed), Culture of marine invertebrate animals: proceedings: 1st Conference on Culture of Marine Invertebrate Animals Greenport. Springer, Boston, MA.

[5] Doyle JJ, Doyle JL 1987 A rapid DNA isolation procedure for small quantities of fresh leaf tissue. Phytochem Bull 19:11–15.

[6] Koren S, Walenz BP, Berlin K, Miller JR, Bergman NH, Phillippy AM 2017 Canu: scalable and accurate long-read assembly via adaptive k-mer weighting and repeat separation. Genome Res 27:722–736. doi:10.1101/gr.215087.116. 28298431 PMC5411767

[7] Kolmogorov M, Yuan J, Lin Y, Pevzner PA 2019 Assembly of long, error-prone reads using repeat graphs. Nat Biotechnol 37:540–546. doi:10.1038/s41587-019-0072-8. 30936562

[8] Laetsch DR, Blaxter ML 2017 BlobTools: interrogation of genome assemblies. F1000Res 6:1287. doi:10.12688/f1000research.12232.1.

[9] Li H 2018 minimap2: pairwise alignment for nucleotide sequences. Bioinformatics 34:3094–3100. doi:10.1093/bioinformatics/bty191. 29750242 PMC6137996

[10] Vaser R, Sović I, Nagarajan N, Šikić M 2017 Fast and accurate de novo genome assembly from long uncorrected reads. Genome Res 27:737–746. doi:10.1101/gr.214270.116. 28100585 PMC5411768

[11] Li H, Durbin R 2009 Fast and accurate short read alignment with Burrows–Wheeler transform. Bioinformatics 25:1754–1760. doi:10.1093/bioinformatics/btp324. 19451168 PMC2705234

[12] Walker BJ, Abeel T, Shea T, Priest M, Abouelliel A, Sakthikumar S, Cuomo CA, Zeng Q, Wortman J, Young SK, Earl AM 2014 Pilon: an integrated tool for comprehensive microbial variant detection and genome assembly improvement. PLoS One 9:e112963. doi:10.1371/journal.pone.0112963. 25409509 PMC4237348

[13] Hunt M, Silva ND, Otto TD, Parkhill J, Keane JA, Harris SR 2015 Circlator: automated circularization of genome assemblies using long sequencing reads. Genome Biol 16:294. doi:10.1186/s13059-015-0849-0. 26714481 PMC4699355

[14] Seemann T 2014 Prokka: rapid prokaryotic genome annotation. Bioinformatics 30:2068–2069. doi:10.1093/bioinformatics/btu153. 24642063

[15] Barbera P, Kozlov AM, Czech L, Morel B, Darriba D, Flouri T, Stamatakis A 2019 EPA-ng: massively parallel evolutionary placement of genetic sequences. Syst Biol 68:365–369. doi:10.1093/sysbio/syy054. 30165689 PMC6368480

[16] Czech L, Stamatakis A 2019 Scalable methods for analyzing and visualizing phylogenetic placement of metagenomic samples. PLoS One 14:e0217050. doi:10.1371/journal.pone.0217050. 31136592 PMC6538146

[17] Simão FA, Waterhouse RM, Ioannidis P, Kriventseva EV, Zdobnov EM 2015 BUSCO: assessing genome assembly and annotation completeness with single-copy orthologs. Bioinformatics 31:3210–3212. doi:10.1093/bioinformatics/btv351. 26059717

[18] Sun C, Wang R-J, Su Y, Fu G-Y, Zhao Z, Yu X-Y, Zhang C-Y, Chen C, Han S-B, Huang M-M, Lv Z-B, Wu M 2017 *Hyphobacterium vulgare* gen. nov., sp. nov., a novel alphaproteobacterium isolated from seawater. Int J Syst Evol Microbiol 67:1169–1176. doi:10.1099/ijsem.0.001780. 28068219

[19] Ruan C-J, Zheng X-W, Wang J, Song L, Zhu Y-X, Du W-B, Lu Z-J, Huang Y, Huang L, Dai X 2018 *Hyphobacterium indicum* sp. nov., isolated from deep seawater, and emended description of the genus *Hyphobacterium*. Int J Syst Evol Microbiol 68:3760–3765. doi:10.1099/ijsem.0.003054. 30516459

[20] Nedashkovskaya OI, Suzuki M, Vysotskii MV, Mikhailov VV 2003 *Reichenbachia agariperforans* gen. nov., sp. nov., a novel marine bacterium in the phylum *Cytophaga*-*Flavobacterium*-*Bacteroides*. Int J Syst Evol Microbiol 53:81–85. doi:10.1099/ijs.0.02128-0. 12656156

[21] Nedashkovskaya OI, Kim SB, Suzuki M, Shevchenko LS, Lee MS, Lee KH, Park MS, Frolova GM, Oh HW, Bae KS, Park H-Y, Mikhailov VV 2005 *Pontibacter actiniarum* gen. nov., sp. nov., a novel member of the phylum “Bacteroidetes,” and proposal of *Reichenbachiella* gen. nov. as a replacement for the illegitimate prokaryotic generic name *Reichenbachia* Nedashkovskaya et al. 2003. Int J Syst Evol Microbiol 55:2583–2588. doi:10.1099/ijs.0.63819-0. 16280531

[22] Cha I-T, Oh Y-S, Park S-J, Park B-J, Lee J-K, Lim C-S, Park A-R, Yoo J-S, Lee D-H, Rhee S-K, Roh D-H 2011 *Reichenbachiella faecimaris* sp. nov., isolated from a tidal flat, and emended descriptions of the genus *Reichenbachiella* and *Reichenbachiella agariperforans*. Int J Syst Evol Microbiol 61:1994–1999. doi:10.1099/ijs.0.026849-0. 20851915

[23] Shi M-J, Wang C, Liu Z-Y, Jiang L-X, Du Z-J 2018 *Reichenbachiella versicolor* sp. nov., isolated from red alga. Int J Syst Evol Microbiol 68:3523–3527. doi:10.1099/ijsem.0.003023. 30231957

